# PROTOCOL: Interventions to improve outdoor mobility among adults with disability

**DOI:** 10.1002/cl2.1280

**Published:** 2022-10-06

**Authors:** Martin Ringsten, Susanne Iwarsson, Eva Månsson Lexell

**Affiliations:** ^1^ Cochrane Sweden, Research and Development Skåne University Hospital Lund Sweden; ^2^ Department of Health Sciences Lund University Lund Sweden; ^3^ Department of Neurology, Rehabilitation Medicine, Cognitive Medicine and Geriatrics Skåne University Hospital Lund‐Malmö Sweden

## Abstract

This is the protocol for a Campbell systematic review. The objectives are as follows: to assess the efficacy of interventions aiming to improve outdoor mobility for people with disability and to explore if the efficacy varies between different populations and different intervention components.

## 1 BACKGROUND

### 1.1 The problem, condition, or issue

Around 15% of the global population, which accounts for around one billion people, live with some form of disability and the number is increasing (WHO, 2020). The International Classification of Functioning, Disability and Health (ICF) is an international conceptual framework used to describe and measure health and disability on both the individual and population level (ICF, 2001). Disability, according to the ICF terminology, is the term for any impairments, activity limitations or participation restrictions and includes all traumatic, congenital, and acquired conditions. The ICF is focusing on health and the consequences of disease, and is closely related to the International Statistical Classification of Diseases and Related Health Problems (ICD‐10), a framework that describes and classifies diseases, disorders, and other health conditions by diagnosis (ICD‐10, 2004). The definition of disability used in the ICF includes many potential causes for disability and can include but is not limited to specific disease classifications.

People living with a disability can experience worse health outcomes (WHO, 2004), experience less participation in the community as well as be part of fewer activities outside the home (Eurostat, 2015). For example, a systematic review exploring unmet long‐term needs reported that 74% of people after stroke perceive at least one or more unmet needs in their life (Chen, 2019). Moreover, people with disability due to dementia have reported decreased out‐of‐home participation and life space outside the home (Margot‐Cattin, 2021).

There are several international and national strategies and policies to guide initiatives to reduce the prevalence and impact of living with disability, for example, the United Nations Convention on the Rights of Persons with Disabilities (CRPD, 2006) and the European Disability Strategy (EU, 2020). In addition, the EU priority areas for people living with disability lists essential areas and goals for further initiatives for people with disability. This includes accessibility to goods and services, participation in public life, activities and community services, and equality to combat discrimination and promote equal opportunities.

Mobility outside the home, or outdoor mobility, is a prerequisite for participation, and includes any way of transportation and mobility in the outdoor environment. This can include using your own body for transportation as walking or using vehicles for transportation as cars, buses, train, tram, bicycles, or scooters (Mollenkopf, 2006). For people living with a disability, using different mobility devices such as a rollator or a manual/electric wheelchair can also facilitate outdoor mobility. Outdoor mobility is necessary to access commodities like grocery stores, pharmacies, or healthcare and to participate in social, physical or other meaningful activities in the community (Rantanen, 2013).

Many individuals living with disability experience limitations to be mobile outside the home (Sainio, 2006). This limitation can be related to environmental constraints, like the presence of stairs, proximity to public transport, or the available information about the current environment. It could also be related to current policy, like regulations and laws or available support from healthcare or social services, for instance having less access to Special Transport Services, often used by people with disability. In addition, outdoor mobility can be limited by individual and personal factors decreasing the capacity to be mobile outside the home and the performance in the current outdoor environment. This can be due to impairments as a result of decreased body and mental functions caused by for example injuries, specific diseases or conditions, or processes associated with aging.

Decreased mobility in life is associated with decreased quality of life (Rantakokko, 2016) and increased social isolation (Schrempft, 2019). Decreased walking performance is also associated to higher all‐cause mortality (Newman, 2006). Consequently, interventions aiming to increase outdoor mobility could impact quality of life and influence activity limitations and participation restrictions in the community and in life overall in a positive way.

The population and target of this review will be people living with any disability that receive interventions aimed to improve outdoor mobility.

### 1.2 The intervention

This review will explore different types of interventions targeted to improve outdoor mobility among people with disability. Interventions can include but are not limited to, educational or psychological/behavioral therapies, physical training interventions, cognitive training interventions or skill training interventions. Interventions can also use and combine several different intervention components within a complex intervention.

For example, a physical training intervention can be aimed to increase balance by training of physical exercises, a skill training intervention can aim to facilitate the use of mental strategies to deconstruct and train specific parts of movement or travel or a cognitive training intervention can aim to reduce fear and avoidance to increase walking outside.

Interventions could be delivered in a variety of settings, including healthcare, the workplace, in the home or in the community, and/or digitally. The delivery of the intervention can also be done in different ways and by different persons, including rehabilitation personnel, social workers, by digital content or by a combination of sources. The interventions can also be delivered in individual sessions, in groups or a combination of the two. Interventions to improve outdoor mobility can vary in intensity and length and include brief educational information in a single session, or include longer and intensive rehabilitation programs containing many intervention components. The interventions can also be tailored to the individual or use a standardized intervention protocol.

**Figure 1:**
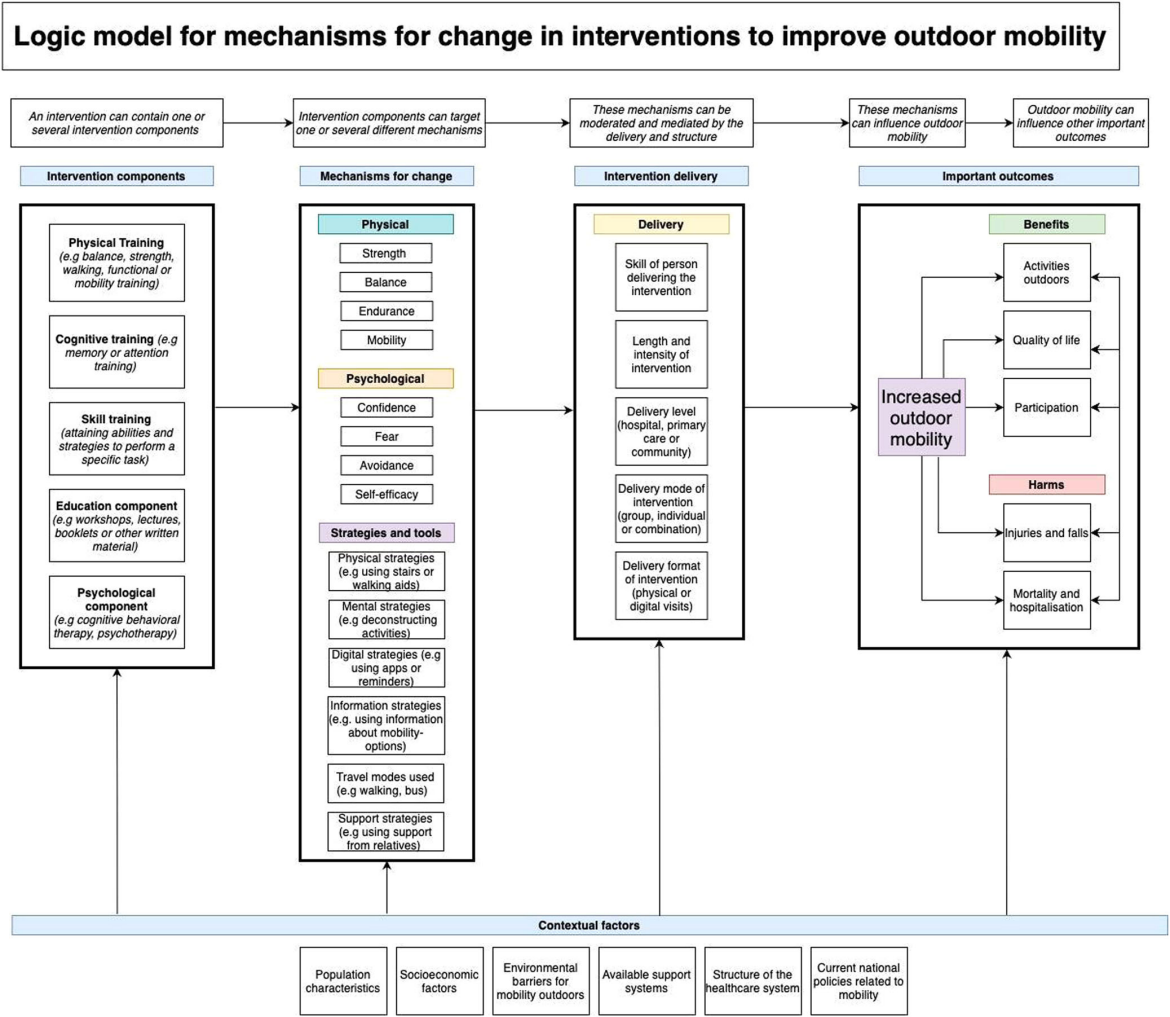
Logic model for interventions to improve outdoor mobility

Interventions that solely target alterations in the environment (e.g., new infrastructure, home adjustments, or increased access to parks) will not be within the scope of the review, see Figure 1 for additional details on included interventions.

### 1.3 How the intervention might work

Outdoor mobility interventions targeting people with disability aim to improve the ability to move, travel, and orient outside the home. In turn, this can influence the number of activities outside the home, increase participation, or improve quality of life. However, outdoor mobility interventions may also lead to harm like falls or injuries or have unforeseen effects which could lead to mortality or hospitalization, which can be related to an increased time walking outdoors and exposure to new environments. An additional unintended harmful effect could be related to interventions making participants more aware of their disability and their actual activity limitations, especially if they have a chronic condition, which could limit acceptance of their disabilities and decrease quality of life.

Outdoor mobility is a complex activity and interventions to improve outdoor mobility can be more or less complex and include one or several intervention components (Craig, 2008). An intervention component can be defined as “a discrete, active element of the intervention that could be implemented independently of other elements” (Lewin, 2017). For example, a physical training component in an intervention can aim to improve outdoor mobility through mechanisms like increasing strength, coordination, or balance, a cognitive training component can aim to improve outdoor mobility through mechanisms like increasing attention or memory to be able to navigate the outdoor environment. In addition, skill training components can aim to improve outdoor mobility through mechanisms like attaining abilities to use tools or strategies for outdoor mobility, education components can try to inform and create knowledge about available travel modes and psychological components can aim to improve outdoor mobility through reducing fear or avoidance for outdoor mobility or increasing self‐efficacy.

Furthermore, the delivery and structure of the intervention could influence the outcomes. For instance, if the intervention is delivered in the home or in a hospital, or if the intervention is based on a digital platform or group‐ or individual‐based (Kubina, 2013) could make a difference. In addition, the number and intensity of treatment sessions or the length of the treatment period could also influence important outcomes. Moreover, important outcomes can be influenced by the skill of the persons delivering the intervention, if it is delivered by interdisciplinary teams and if it requires certain interventionist skills. In addition, outcomes could be affected if the intervention includes motivational components or platforms that could facilitate actual outdoor mobility.

The potential effects of interventions that aim to improve outdoor mobility can differ depending on the specific target population, and the context and outdoor environment the population will be mobile in. In addition, national policies and available support systems for outdoor mobility as well as the structure of the healthcare system could influence the effects of the interventions.

### 1.4 Why it is important to do this review

Published RCTs using interventions aimed at improving outdoor mobility are quite scarce. Most commonly they are conducted within one specific population, for example, people after stroke (Logan, 2014), people with musculoskeletal injuries (Magaziner, 2019), or older adults (Rantanen, 2020).

Systematic reviews evaluating interventions for people with disabilities—a broad population including but not limited to different diseases and conditions—are also scarce. For example, two systematic reviews have explored interventions to support community participation for people with disability (Gross, 2020) and interventions to increase motor skills (Bishop, 2018). Recently published trials as well as ongoing trials (Chang, 2020), have the potential to be included in this current review and further deepen the evidence base for outdoor mobility interventions.

Previously published systematic reviews of outdoor mobility interventions were limited to walking as a way of outdoor mobility but left out other modes of transport like busses, trains, or bikes. For example, a previous systematic review evaluated interventions for improving community ambulation for people with stroke (Barclay, 2015). This review focused specifically on interventions improving walking in the community and excluded trials exploring other modes of mobility and travel. The literature search was conducted in 2014 and only five trials presenting a diverse set of outcome measures could be included, which resulted in that no conclusions could be drawn from the limited sample. An upcoming systematic review (Barclay, 2020) will explore interventions to improve walking in the community but will only include populations of older adults, regardless of disability. Furthermore, a previous systematic review explored factors in relation to outdoor mobility for people with multiple sclerosis (van der Feen, 2020) but did not locate any randomized controlled trials (RCT) to evaluate the efficacy of interventions to improve outdoor mobility.

In summary, previous systematic reviews have explored different modes of outdoor mobility for specific populations based on diagnoses but have not found sufficient published evidence to be able to draw any conclusions. To our knowledge, no previous systematic review has specifically explored whether interventions to improve outdoor mobility are effective for people with disabilities.

Furthermore, to our knowledge, no systematic review has explored differences in effect estimates between different classifications of disease or type of disability or explored the potential intervention mechanisms of action or intervention components which could have an influence on important outcomes. Thus, previous systematic reviews have not been able to guide policymakers or clinicians in decisions about implementation of interventions to promote outdoor mobility. There is uncertainty related to in what populations interventions to improve outdoor mobility have been evaluated. Moreover, previous reviews have not been able to provide guidance on potentially effective components or modes of delivery for the design of future interventions aimed at improving outdoor mobility for adults with disability.

The proposed review, *with the aim to evaluate the efficacy of outdoor mobility interventions*, has the potential to guide policymakers and clinicians in making evidence‐informed decisions toward the goal of increasing accessibility, participation, and activities in the community and to work toward equal opportunities and increase health and quality of life for people living with disability. The conclusions of this review could also inform the development and implementation of future interventions designed to improve outdoor mobility for people with various disabilities.

## 2 OBJECTIVES

1. To assess the efficacy of interventions aiming to improve outdoor mobility for people with disability.
2. To explore if the efficacy varies between different populations and different intervention components.

## 3 METHODS

### 3.1 Criteria for considering studies for this review

#### 3.1.1 Types of studies

Eligible study designs to be included in the review are RCTs where participants have been randomly allocated to an intervention or control group. To minimize the risk of bias of the effect estimates we will exclude cluster randomized trials, cross‐over trials, and quasi‐randomized trials in this review. We will exclude non‐randomized studies, observational studies, and case reports.

The decision to restrict the study design to only randomized controlled studies of interventions was done because it will minimize the risk of any bias influencing the effect estimates of these interventions. Randomization is the only approach to prevent systematic differences for baseline characteristics of participants in the intervention and control group in terms of both known, unknown, or unmeasured confounders (Higgins et al., 2019). We acknowledge the trade‐off between our restrictive criteria for study designs—which could lead to including fewer studies within the review—compared to a broader criterion including more studies but subsequently with a higher risk of bias. This choice was supported by the knowledge that studies using randomized study designs examining interventions to improve outdoor mobility are available in the evidence base (see Background) thus less bias will possibly be introduced into the review and the effect estimates of the outcomes of the intervention.

#### 3.1.2 Types of participants

Participants eligible for inclusion are all adults, aged 18 or above, living with one or multiple disabilities. All types of disabilities will be eligible and can include physical, mental, cognitive or sensory disabilities.

For example, this can include populations living with:

- Diseases of the musculoskeletal system or connective tissue, like osteoarthritis and fractures.
- Mental, behavioral or neurodevelopmental disorders, like depression and autism.
- Diseases of the nervous system, like multiple sclerosis and stroke.
- Diseases of the visual system, like vision impairments.
- Injury, poisoning, or certain other consequences of external causes, like traumatic amputations.
- Diseases of the circulatory system, like ischemic heart disease.
- Diseases of the respiratory system, like chronic obstructive pulmonary disease.
- Diseases related to aging, like pathological processes which lead to the loss of adaptation and progress in older ages.

We will include participants in all types of settings and will not limit the inclusion to any specific demographics.

We will exclude studies where more than 50% of the population is below the age of 18. We will also exclude studies where more than 50% of the population are not described in terms of having a disability defined as impairments in a person's body structure or mental functioning, activity limitations or participation restrictions. As we will only include studies where participants with disability have been randomly assigned to either an intervention or a control group, both study arms will include people representing the same population, that is we will not compare samples with disability with samples without disability.

#### 3.1.3 Types of interventions

This review will include interventions that aim to improve outdoor mobility for people with disability. It could include for example educational interventions, physical training interventions, cognitive or behavioral training interventions, or skill training interventions. Interventions can be delivered on their own or in packages of several interventions by a single healthcare provider or a multidisciplinary team.

The included interventions can be delivered in the community, for example within healthcare, the workplace, within the home, digitally, or by groups in the community. The interventions can be delivered directly related to an injury, but also several years after having received a diagnosis, commonly for people living with a progressive disorder. Interventions aimed solely for health promotion and/or primary prevention will be excluded. For example, interventions aiming to prevent a future decline in outdoor mobility for older adults who currently do not have limited mobility outdoors will be excluded.

No restrictions will be placed on duration, intensity, and way of delivery of the intervention, as it can vary due to different needs for the participants and resources for delivery in the trial context. We will extract and describe the duration, intensity, and frequency for all included interventions.

The focus of the intervention to improve outdoor mobility has to be clearly described as an aim or purpose of the intervention to be included. Such descriptions will be extracted and presented for all included interventions. Interventions without an aim or purpose to improve outdoor mobility will be excluded, for example, interventions aiming to only improve physical activity levels or walking speed for the participants. In addition, interventions with a focus to only improve mobility indoors or personal ADL will be excluded.

Environmental components can be included as a part of an intervention only if they are part of multi‐component interventions that include components aimed at the capacity or performance of the participants as well. Such interventions can include home modifications in combination with skill training aiming to increase the performance to travel outside.

Interventions that solely target alterations in the environment will be excluded from this review. This includes interventions targeting infrastructure, changes in organizations, routines or policies, implementation of ramps or lifts, implementation of digital support systems, only providing mobility aids, adjustments in the home or increased access to training sites or parks.

We will include two comparisons in the review:

1. Outdoor mobility intervention compared with a control intervention not expected to improve outdoor mobility. The control intervention include:
  - A usual care‐control group as stated by the trial authors.
  - A wait‐list control group.
  - A control group receiving no intervention.
  - An attention control group, that is, a control intervention not aimed to improve outdoor mobility.
2. An outdoor mobility intervention compared to another outdoor mobility intervention. This can include interventions containing different components or different forms of delivery, for example, individual versus group delivery, digital versus physical delivery, or including balance training in an educational intervention to improve outdoor mobility in only one group.

#### 3.1.4 Types of outcome measures

We will extract outcome data from studies using both self‐reported and observer‐reported outcome measures. The outcome measures can be reported by digital means (e.g. activity monitors and GPS monitors), by the use of journals, questionnaires, or other scales or assessments.

##### Important outcomes

We will include any of the six following important outcomes in the review:

1. **Activity outside the home**

Activity outside the home (defined according to ICF as “the execution of a task or action by an individual”) can be measured in continuous outcome measures, for example:

• Number of times going outside the home
• Journeys outside the home
• Activities outside of the home
• Steps taken outside the home
• Specific assessment tools such as the “Life Space assessment Questionnaire” (Baker, 2003).

The outcome measures will be extracted and categorized according to at what time after starting the intervention they were collected, defined as short‐term (≤6 months) or long‐term (≥7 months).

2. **Engagement in everyday life activities**

Engagement in everyday life activities (defined according to Kielhofner and colleagues (Taylor, 2017) as “engagement in work, play, or activities of daily living that are part of one's socio‐cultural context and that are desired and/or necessary to one's well‐being”) can be measured by dichotomous outcome measures, for example, where study participants have answered “yes” or “no” to questions such as:

- “Can you do what you want to do in your life?”
- “Are you able to engage in valuable activities?”
- “Do you get out of the house as much as you would like?”
- “Do you avoid certain activities outdoors?”

The outcome measures will be extracted and categorized according to at what time after starting the intervention they were collected, defined as short‐term (≤6 months) or long‐term (≥7 months).

3. **Participation**

Participation (defined according to ICF as “involvement in a life situation”) can be measured in continuous outcome measures, for example:

- Impact of Participation and Autonomy Questionnaire (IPA) (Cardol, 2001)
- WHO Disability Assessment Schedule 2.0 (WHODAS 2.0) (Ustun, 2010)
- Participation Objective Participation Subjective Instrument (POPS) (Brown, 2004)
- Participation measure for post‐acute care (PM‐PAC) (Gandek, 2007)
- Rating of Perceived Participation (ROPP) (Sandström, 2007)
- The Participation Scale (Van Brakel, 2006)
- The Keele Assessment of Participation (KAP) (Wilkie, 2005)
- Other disease‐specific measurements of Participation, if the content and construct is similar to the generic measurements

The outcome measures will be extracted and categorized according to at what time after starting the intervention they were collected, defined as short‐term (≤6 months) or long‐term (≥7 months).

4. **Health‐related quality of life**

Health‐related quality of life (defined according to WHO, 2012 as “individuals’ perceptions of their position in life in the context of the culture and value systems in which they live and in relation to their goals, expectations, standards, and concerns”) can be measured in continuous outcome measures, for example:

- SF36 (Ware, 2007)
- SF12 (Ware, 1996)
- EQ. 5D (Rabin, 2001)
- WHOQOL (WHO, 2001)
- Health Utilities Index (HUI) (Furlong, 2001)
- CDC HRQOL‐4 (Moriarty, 2003)
- Quality of Life Questionnaire for Older People (OPQOL‐brief) (Bowling, 2013)
- Other disease‐specific measurements of HRQOL, if the content and construct is similar to the generic measurements.

The outcome measures will be extracted and categorized according to at what time after starting the intervention they were collected, defined as short‐term (≤6 months) or long‐term (≥7 months).

5. **Major adverse events**

Major adverse events can be based on dichotomous or continuous outcome measures, and will include:

- Mortality
- Hospitalization
- Injuries requiring medical attention in a hospital.

The outcome measures will be extracted and categorized according to at what time after starting the intervention they were collected, defined as short‐term (≤6 months) or long‐term (≥7 months).

6. **Minor adverse events**

Minor adverse events can be based on dichotomous or continuous outcome measures, for example:

- Falls
- Injuries not requiring hospitalization
- Other possible adverse events reported in the included studies.

The outcome measures will be extracted and categorized according to at what time after starting the intervention they were collected, defined as short‐term (≤6 months) or long‐term (≥7 months).

Falls leading to hospitalization or injuries requiring medical attention will be classified as Major Adverse Events. Other falls will subsequently be classified as a Minor Adverse event, which has been estimated to be approximately 98.8% of all falls in a hospital setting (Kobayashi, 2017).

#### 3.1.5 Duration of follow‐up

We will include all duration of follow‐up, but will be separated into two different categories, that is, short‐term (6 months or less) and long‐term (7 months or more) after starting the intervention.

#### 3.1.6 Types of settings

We will include interventions delivered in any setting, for example in hospitals, clinics, the workplace, within the home, digitally or by groups or organizations in the community. We expect that the different settings can have varying impacts on the estimated effects of the intervention and will explore this heterogeneity within a subgroup analysis.

### 3.2 Search methods for identification of studies

A search strategy has been developed involving three information specialists with experience in conducting search strategies for systematic reviews according to the Cochrane Methodology in close collaboration with the review authors.

We will search:

- MEDLINE (EbscoHost)
- CINAHL Complete via EBSCOhost
- Embase via Embase.com
- Cochrane Central Register of Controlled Trials (CENTRAL) via Wiley Cochrane Library
- AMED (The Allied and Complementary Medicine Database) via EBSCOhost
- PsycInfo via EBSCOhost
- PEDro (Physiotherapy Evidence Database)
- ERIC (Education Resource Information Centre) via EBSCOhost
- Scopus
- Web of Science Core Collection: Conference Proceedings Citation Index‐ Science (CPCI‐S), and Conference Proceedings Citation Index‐ Social Science & Humanities (CPCI‐SSH)

We will also search for ongoing or recently completed studies at:

- Clinicaltrials.gov
- The World Health Organization International Clinical Trials Registry Platform (WHO ICTRP)

An updated search will be done when the screening and data extraction is completed. We will not apply any language restrictions. If help with translation is needed we will primarily reach out to the Cochrane network for support.

#### 3.2.1 Electronic searches

The search strategy for MEDLINE via EBSCOhost is available in Supporting Information: Appendix 1. Search strategies for other databases and depositories will be based on the MEDLINE strategy and will be included in full in the upcoming review.

#### 3.2.2 Searching other resources

An additional hand search will be made in the reference list of systematic reviews identified in our literature search, together with a manual search of the reference lists of the studies included in the review.

### 3.3 Data collection and analysis

#### 3.3.1 Description of methods used in primary research

We expect primary research to use a variety of methods for determining the efficacy of interventions to improve outdoor mobility, and some of these studies will use a randomized controlled design. The interventions will differ in their delivery and will have different active components. We anticipate that most comparison groups will be categorized as treatment as usual or no intervention. The studied populations with disability will vary but we expect that a large proportion of the populations in the included studies will be people with disability after stroke.

#### 3.3.2 Selection of studies

Two reviewers will independently screen titles and abstracts and exclude reports which do not match our inclusion and exclusion criteria. The Covidence software will be used for the screening process. Any disagreements will be solved by discussion together with a third author. If insufficient information to assess eligibility is available in the title or abstract the report will be assessed in full text.

Two reviewers will independently assess the full‐text versions of the reports in Covidence and disagreements will be solved together with a third reviewer by consensus. If the eligibility of the report is still uncertain attempts will be made to contact the primary investigators of the report for clarification.

#### 3.3.3 Data extraction and management

Two reviewers will independently extract data from the included reports. The data extraction form (Supporting Information: Appendix 2) will be piloted by the reviewers in Covidence and revised before starting data extraction. Disagreements in data extraction will be solved together with a third reviewer by consensus. The analysis are planned to be conducted in RevMan Web.

#### 3.3.4 Assessment of risk of bias in included studies

Two reviewers will independently assess the risk of bias in each study outcome using “Risk of bias in randomised trials (RoB2)” tool (Higgins et al., 2021). Disagreements in the risk of bias‐evaluations will be solved together with a third reviewer by consensus.

We will assess risk of bias for each study outcome using the five domains from RoB2 (Cochrane Handbook).

- Domain 1: Bias arising from the randomization process
- Domain 2: Bias due to deviations from intended interventions
- Domain 3: Bias due to missing outcome data
- Domain 4: Bias in measurement of the outcome
- Domain 5: Bias in selection of the reported result

For each domain, a series of signaling questions (yes, probably yes, no information, probably no, no) will guide the judgment of risk of bias (low risk, some concerns, and high risk). We will include text alongside these judgments to provide supporting information for our decisions. The risk of bias judgment will be presented alongside the effect estimates in the forest plot of a meta‐analysis.

#### 3.3.5 Measures of treatment effect

Statistical analyses will be done using Review Manager (RevMan Web, 2020) and will summarize the data in a meta‐analysis if they are sufficiently homogeneous, both clinically and statistically. If a meta‐analysis is not feasible the results will be presented narratively, if applicable, within a forest plot.

Dichotomous data will be analyzed using relative risk ratio (RR) together with 95% confidence intervals (CI).

Continuous data will be converted to standardized mean difference (SMD) together with 95% confidence intervals for analysis. If data in the reports are not presented in means, standard deviations or effect sizes, methods suggested in the Cochrane Handbook will be used to calculate the standardized mean difference if possible.

If both adjusted and non‐adjusted effect estimates are reported in the same study, we will extract data from the adjusted estimate if sufficient data is reported to use this effect estimate in a pooled analysis.

#### 3.3.6 Unit of analysis issues

Studies with multiple outcome measurement time‐points will be extracted and categorized according to what time after commencing treatment they were collected, that is, short‐term (≤6 months) or long‐term (≥7 months). If several time points are reported within one of the categories we will use and extract the time point with the longest follow‐up.

If a study with three or more intervention arms fits our inclusion criteria for more than one intervention or comparison only include the intervention arm that is most closely aligned with the study aim and inclusion criteria will be included. In addition, we will perform a sensitivity analysis exploring the potential impact of our choice.

#### 3.3.7 Criteria for determination of independent findings

The review will focus on each eligible study and not on each report of a study. Different reports from the same study population will be handled as one study.

If several outcome measures are used to measure the same or a similar outcome in an included study, we will only extract one of the outcome measures as described in the Cochrane Handbook (Cochrane Handbook). All outcomes will be transparently reported in the “Characteristics of included studies” and sensitivity analysis will be done using all outcomes reported in the outcome category to test the robustness.

To mitigate any risk of outcome‐driven choices when several outcome measures are reported within the same outcome category within a study, we will extract outcomes based on a hierarchy inspired by examples in the Cochrane Handbook (Cochrane Handbook).

1. When possible, we will extract the outcome measure that is the primary outcome in each included study.
2. When possible, we will extract the outcome used in the sample size calculation within the study.
3. If the above is not possible, we will extract the outcome from the list of outcome measures presented in the “Important outcome” section in the protocol. If several of the outcome measures are presented, the measures closest to the top of the list of outcome measures will be extracted.

In addition, we will consult a statistician specialized in psychometrics for guidance if two measurements within the same outcome domain are identified and reported within a study.

A sensitivity analysis will be conducted when several outcomes are reported within the same domain to explore if our choice of outcome has an impact on the results, see the Sensitivity Analysis section.

If only a part of a measurement or a subscale is presented (e.g., only the Social Function‐domain in SF36) and full data for this outcome measure is not available through contact with study authors, we will explore if the part or subscale is overlapping with the construct of the intended outcome in a meaningful way. If not, this outcome measure will be excluded from the analysis.

#### 3.3.8 Dealing with missing data

Missing data will be identified in the data extraction process of the included full‐text reports. We will attempt to contact the primary investigators of the report to obtain missing data. If missing data is not able to be obtained from the primary investigators, or calculated based on guidance from the Cochrane Handbook which prevents calculating a SMD or a RR, the report will be excluded from a meta‐analysis and the Summary of Findings‐table (SoF‐table) but will be presented separately in the result section. The Missingness of outcome data will be handled according to the guidance provided in the Cochrane Handbook for data extraction and Risk of Bias judgments.

#### 3.3.9 Assessment of heterogeneity

To assess the heterogeneity of the effect estimates we will use the *I* ^2^ statistic together with a visual assessment of the forest plots.

We will use the thresholds, as reported in the Cochrane Handbook, as a rough guide to the interpretation of the *I* ^2^ statistic:

- 0%–40%: might not be important;
- 30%–60%: may represent moderate heterogeneity;
- 50%–90%: may represent substantial heterogeneity;
- 75%–100%: considerable heterogeneity.

The potential heterogeneity will be further explored in subgroup analysis and sensitivity analysis and will support the decision of whether conducting a meta‐analysis is appropriate or not.

#### 3.3.10 Assessment of reporting biases

We will create and examine a funnel plot to explore the possibility of small‐study biases. In interpreting the funnel plot, we will examine the different reasons possible for funnel plot asymmetry as outlined in section 10.4 of the Cochrane Handbook, and relate this to the results of the review. If we are able to pool more than 10 trials, we will undertake formal statistical tests to investigate funnel plot asymmetry and follow the recommendations in section 10.4 of the Cochrane Handbook. To assess outcome reporting bias, we will check trial protocols against published reports. For studies published after 1 July 2005, we will screen the Clinical Trial Register at the International Clinical Trials Registry Platform of the World Health Organization (http://apps.who.int/trialssearch) for the trial protocol.

#### 3.3.11 Data synthesis

Pooling the included studies in a meta‐analysis will be done if appropriate, considering multiple studies reporting the same outcome and the availability of outcome data to enable data synthesis.

Based on guidance provided in the Cochrane Handbook (Cochrane Handbook), the decision to conduct a meta‐analysis will be based on the level of heterogeneity of included interventions, included populations and based on the effect estimate in our comparisons and the predefined subgroups. For example, if the clinical heterogeneity is considered too large and a statistical synthesis of effect estimates from very different studies could lead to misleading effect estimates, no meta‐analysis will be conducted.

We expect a large variation between different interventions included in this review and due to the heterogeneity we will, if applicable, use a random‐effects model. The heterogeneity will be further explored within subgroup analyses and sensitivity analyses. If we judge meta‐analysis to be inappropriate according to guidance from the Cochrane Handbook (Cochrane Handbook), we will analyze and interpret individual studies separately in a narrative synthesis.

#### 3.3.12 Subgroup analysis and investigation of heterogeneity

Subgroup analyses will be conducted in three different areas based on:

1. Population characteristics. Subgroup analysis of population characteristics will be grouped according to diagnose group as defined in the included studies. For example: stroke, dementia, fractures, osteoarthritis, and older adults.
2. Risk of Bias. Subgroup analysis of risk of bias judgments will be grouped according to the different overall risk of bias judgments: Low risk of bias versus high risk of bias or some concern.
3. Intervention components and intervention characteristics. Due to the number of subgroup analyses planned in this area, they will be considered exploratory in nature. These subgroups will consist of:
  3.1. Different intervention components:
    - Physical training component (e.g., balance training, resistance training, walking training)
    - Cognitive training component (e.g., memory and attention training)
    - Skill training component (Skill training (e.g., attaining abilities needed to perform a task, which can be more or less complex. For example, learning to use an app or mobility aid, learning new modes of doing a certain task or training to perform all the parts of a specific activity. For instance, perform all steps included in the travel chain when traveling with public transport)
    - Educational component (e.g., seminars, presentations, workshops or written material)
    - Psychological component (e.g., Cognitive Behavioral Therapy or Psychotherapy)
    - A combination of different intervention components that together constitute the study intervention.
  3.2. Different control interventions
    - Usual care‐control as stated by the trial authors, wait‐list control and control group receiving no intervention
    - Attention control group (i.e., an active control intervention not aimed to improve outdoor mobility)
  3.3. Different intervention areas (e.g., intervention components including changes in the environment):
    - Including environmental components in the intervention together with intervention to increase capacity and performance.
    - Including only interventions aimed to increase capacity and performance.
  3.4. Different intervention delivery:
    - Group‐based
    - Individual‐based
    - Combination of group and individual
  3.5. Different intervention duration:
    - Subgroup analysis based on the length of the intervention period. The subgroups will be defined after data collection and will be regarded as exploratory in nature.
  3.6. Different intervention mobility modes:
    - Walking (e.g., with or without walking aids)
    - Buss or tram
    - Train
    - Electric vehicles (e.g., mobility scooters, e‐bikes)
    - Combination of mobility modes
  3.7. Different intervention settings:
    - Community/outpatient (e.g., intervention delivered in your own home environment, in primary care, or by groups in the broader community)
    - Hospital
    - Nursing home

#### 3.3.13 Sensitivity analysis

Sensitivity analyses will be conducted to explore the potential impact the methods chosen and used in the review could have on our conclusions. Exploration of the potential impact is planned to be done by:

- Removing studies using “per protocol” or “as treated” analysis (adherence to intervention) instead of intention to treat (assignment to intervention).
- Removing studies having a high risk of bias due to missing outcome data (attrition bias).
- Including the reported outcomes for the second active intervention arm in studies having more than two arms.
- Including the second reported outcome measures in studies reporting multiple outcome measures for the same outcome.
- Removing outcome measures being reported by subscales or parts of measurements, which is applicable when full outcome measures have not been reported in a study.
- Removing studies where environmental interventions are combined with interventions to increase individuals’ capacity and performance.
- Removing studies using balance training as an intervention for the outcome of adverse events, including falls.

#### 3.3.14 Treatment of qualitative research

We do not plan to include qualitative research.

#### 3.3.15 Summary of findings and assessment of the certainty of the evidence

Two reviewers will independently rate the certainty of the body of evidence for the different included outcomes. We will use the GRADE system to rank the certainty of the evidence using the GRADEprofiler Guideline Development Tool software (GRADEpro) following the guidelines in the Cochrane Handbook and GRADE Handbook.

The GRADE approach includes five domains: study limitations (risk of bias), unexplained heterogeneity and inconsistency of effect, imprecision, indirectness, and publication bias to assess the certainty of the body of evidence for each outcome.

The GRADE system uses the following criteria for determining the certainty of evidence:

- High: we are very confident that the true effect lies close to that of the estimate of the effect.
- Moderate: we are moderately confident in the effect estimate; the true effect is likely to be close to the estimate of effect, but there is a possibility that it is substantially different.
- Low: our confidence in the effect estimate is limited; the true effect may be substantially different from the estimate of the effect.
- Very low: we have very little confidence in the effect estimate; the true effect is likely to be substantially different from the estimate of effect.

The GRADE system uses study design as a marker of quality. RCTs are considered to be a higher certainty of evidence but can be downgraded for important limitations.

Factors that may decrease the certainty in a body of evidence are:

- Serious or very serious study limitations (risk of bias)
- Important or serious inconsistency of results
- Some or major indirectness of evidence
- Serious or very serious imprecision
- Probability of publication bias

We plan to include a total of two “Summary of findings” (SoF) tables to present the main findings of the review which will follow the structure as described in the (Cochrane Handbook, Chapter 14). We will include key information concerning the certainty of evidence, the magnitude and confidence interval of the effect of the interventions examined, and the sum of available data on the outcomes.

1. Summary of Findings Table 1 will summarize the six outcomes measured in the short term (≤6 months) for outdoor mobility intervention compared with a control intervention.
2. Summary of Findings Table 2 will summarize the 6 outcomes measured in the long term (≥7 months) for outdoor mobility intervention compared with a control intervention.

In addition, for each population group defined as a separate diagnose group in our subgroup analysis we will include two Summary of findings tables, one for short term (≤6 months) and one for long term (≥7 months), including the six defined outcomes included in this review. We plan to do this to more accurately describe the certainty of evidence for each population. This could increase the usability of the review for policy‐makers and treatment providers who make decisions only for certain populations with disability.

## CONTRIBUTIONS OF AUTHORS

- Content: Martin Ringsten, Susanne Iwarsson, Eva Månsson‐Lexell
- Systematic review methods: Martin Ringsten, Matteo Bruschettini
- Statistical analysis: Martin Ringsten
- Information retrieval: Matthias Bank

## DECLARATIONS OF INTEREST

EML and SI has previously developed intervention, which has been evaluation in a feasibility study, within the topic of this review. Due to the inclusion criteria of this review, this study will not be included in the upcoming review.

MR, EML, and SI will use the conclusions from this review in developing a novel intervention to improve outdoor mobility. An update of this review might include this study, which will have to adhere to the same methodology as presented in this protocol.

## PRELIMINARY TIMEFRAME

The systematic review is planned to be submitted 1 year from submission of the review protocol, that is, June 2022 the latest.

## PLANS FOR UPDATING THIS REVIEW

Updates of this review are planned to be carried out at least every 4 years. The main responsibility to initiate an update will be the contact author of the review.

## SOURCES OF SUPPORT

**Internal sources**

• Cochrane Sweden, Sweden
  Staff within Cochrane Sweden will be available for support for methodological questions.
• Lund University Library, Sweden
  The library at Lund University is available to provide support for conducting, running and reporting the search strategy used in the review.

**External sources**

• FORMAS Grant, Sweden

## Supporting information

Supporting information.
