# Supplementary material for: PROTOCOL: Interventions to improve outdoor mobility among adults with disability
Source: Campbell Syst Rev. 2022 Oct 6;18(4):e1280. doi: 10.1002/cl2.1280 (PMC9538711; doi:10.1002/cl2.1280)
Supplement: Supplementary file 1 — Supporting information. [file CL2-18-e1280-s001.docx]

**Appendices**

**1 Appendix 1: Search Strategy for MEDLINE**

| S1 AND S2 |
| --- |
| PT ( randomized control trial OR controlled clinical trial ) OR AB ( randomized OR placebo OR randomised OR randomly OR rct OR trial OR groups ) OR TI ( randomized OR placebo OR randomised OR randomly OR rct OR trial OR groups ) NOT ( (MH "Animals+") NOT MH humans ) |
| ( (community OR outdoor* OR (outside N3 home) OR "out of home") N3 (ambulat* OR transportation OR walk* OR mobility OR travel*) ) OR ( "life space mobility" OR "life space assessment" OR "walking capacity" OR "walking ability" OR "outdoor activit*" OR "outside activit*" OR (participation N3 outdoor*) ) OR (MM "Mobility Limitation") |

**2 Appendix 2: Data extraction form**

Appendix 2: Data extraction form

**1. General information about the included trial**

- 1.1. Study ID in Covidence. *Example: “#12345”.*
- 1.2. First author of the main study and year of publication. *Example: “Logan 2014”.*
- 1.3. Name of study. *Example: “Outdoor mobility intervention for disability: a randomized controlled trial”.*
- *1.4. Funding source of the trial. Example: "The NHS research and development department"*
- *Declaration or conflicts of interest.*

**2. Population in the included trial.**

- 2.1. Specific population or condition. *Example: “Stroke”, “Dementia”, “Osteoarthritis”, “Older adults” etc.*
- 2.2. Age of participants. *Description of mean age and Standard Deviation (SD) for included participants. If mean is not available, use Median and IQR. Example: Mean 71 (5).*
- 2.3. Gender of participants. *Example: “55% Women”*
- 2.4. Country. *Example: “Sweden”, “UK”, “Taiwan” etc.*
- 2.5. Recruitment setting. *Example: “Hospital”, “Community” etc.*

**3. Interventions in the included trial.**

- 3.1. Name of intervention. *Example: “GetOut”, “BusTrips”, “Walking-In-Community" or “resistance training”.*
- 3.2. Year when the intervention was initiated and conducted. *Example: “2012”.*
- 3.3. Intervention setting. *Example: “Community”, “hospital”, “primary care” or “digital”.*
- 3.4. Intervention delivery. *Example: “Individual”, “Group”.*
- 3.5. Intervention components. *Example: “Physical training”, “Cognitive training”, “Skill training”, “Education”, “Phycological training”.*
- 3.6. Type of intervention components related to travel. *Example: “Walking”, “bus”, “tram”, “car”, “mobility aids”, “bicycle”, “electronic travel modes”, or several travel components.*
- 3.7. Tailoring of the intervention. *Was the intervention delivered as a strict program or was it possible to tailor the intervention to the participants? Example: “The intervention was personalized to the participants in accordance to their set goals of activity”.*
- 3.8. Duration of intervention in number of sessions. *Described as mean with SD or median with IQR. Example: “10 sessions, (4 SD)”*
- 3.9. Intensity of intervention in frequency and length of intervention sessions.  *Time of average mean session in minutes and number of intervention sessions per week. Example: “60-minute sessions”*
- 3.10. Duration of intervention in the length of the active intervention period. *Described as mean with SD or median with IQR in weeks from start of intervention to end of intervention.  Example: “10 weeks”*
- 3.11. Credentials of the treatment provider. *Example: “Physiotherapist”, “occupational therapist”, “nurses”, “social workers”, “psychologists”, “medical practitioner”, “multiple treatment providers” or “unclear/not reported”.*
- 3.12. Comparison intervention. *Example: “Wait-list control”, “treatment as usual”, “attention control” or “no treatment”. Describe additional details about the comparison, for example extent of the control intervention if available in the article.*

**4. Outcomes of interest included in the trial**

- 4.1. Reported outcomes of interest. *Example: “Activity outside the home”, “health-related quality of life”, “participation”, or “adverse events”.*
- 4.2. Specific measure of the outcome. *Example: “SF36”, “journeys outside the home” or “number of falls”.*
- 4.3. Outcome measure data report. *Example: “Self-reported”, “carer-reported”, “assessor reported”.*
- 4.4. Time-points of collection of outcome measures. *Specific time-points of reported outcome measures based on time from the beginning of the intervention. Example: Outcomes reported at 1, 3 and 12 months.*

**5. Methods used in the included trial**

- 5.1. Number of participants in each group at randomization. *Example: “100 participants in the intervention group and 99 participants in the control group at randomization”.*
- 5.2. Number of participants at *the* analysis of outcomes. *Example: “At 9 month follow up 80 participants reported outcomes in the intervention group and 50 in the control group”.*
- 5.3. Analysis based on Intention-to-treat or per protocol. *Example. “Analysis carried out as intention-to-treat.”*
